# Personalized surgical informed consent with stereoscopic visualization in neurosurgery—real benefit for the patient or unnecessary gimmick?

**DOI:** 10.1007/s00701-023-05512-x

**Published:** 2023-02-28

**Authors:** Nicolas Hertzsprung, Kiril Krantchev, Thomas Picht, Anna L. Roethe, Kerstin Rubarth, Josch Fuellhase, Peter Vajkoczy, Güliz Acker

**Affiliations:** 1 Department of Neurosurgery, Charité – Universitätsmedizin Berlin, Corporate Member of Freie Universität Berlin and Humboldt-Universität Zu Berlin, Charitéplatz 1, 10117 Berlin, Germany; 2 Cluster of Excellence: “Matters of Activity. Image Space Material”, Humboldt-Universität Zu Berlin, 10099 Berlin, Germany; 3 Institute of Biometry and Clinical Epidemiology, Charité – Universitätsmedizin Berlin, Corporate Member of Freie Universität Berlin and Humboldt-Universität Zu Berlin, Charitéplatz 1, 10117 Berlin, Germany; 4 Berlin Institute of Health at Charité – Universitätsmedizin Berlin, Charitéplatz 1, 10117 Berlin, Germany; 5 Institute of Medical Informatics, Charité – Universitätsmedizin Berlin, Corporate Member of Freie Universität Berlin and Humboldt-Universität Zu Berlin, Charitéplatz 1, 10117 Berlin, Germany; 6 BIH Academy, Clinician Scientist Program, Berlin Institute of Health at Charité – Universitätsmedizin Berlin, Charitéplatz 1, 10117 Berlin, Germany

**Keywords:** 3D, Stereoscopic, Neurosurgery, Surgical informed consent, Patient education

## Abstract

**Background:**

Informed consent of the patient prior to surgical procedures is obligatory. A good and informative communication improves patients’ understanding and confidence, thus may strengthen the patient-doctor relationship. The aim of our study was to investigate the usefulness of additional stereoscopic visualization of patient-specific imaging during informed consent conversation.

**Methods:**

Patients scheduled for a brain tumor surgery were screened for this study prospectively. The primary exclusion criteria were cognitive or visual impairments. The participants were randomized into two groups. The first group underwent a conventional surgical informed consent performed by a neurosurgeon including a demonstration of the individual MRI on a 2D computer screen. The second group received an additional stereoscopic visualization of the same imaging to explain the pathology more in-depth. The patients were then asked to fill in a questionnaire after each part. This questionnaire was designed to assess the potential information gained from the patients with details on the anatomical location of the tumor as well as the surgical procedure and possible complications. Patients’ subjective impression about the informed consent was assessed using a 5-point Likert scale.

**Results:**

A total of 27 patients were included in this study. After additional stereoscopic visualization, no significant increase in patient understanding was found for either objective criteria or subjective assessment. Participants’ anxiety was not increased by stereoscopic visualization. Overall, patients perceived stereoscopic imaging as helpful from a subjective perspective. Confidence in the department was high in both groups.

**Conclusion:**

Stereoscopic visualization of MRI images within informed consent conversation did not improve the objective understanding of the patients in our series. Although no objective anatomical knowledge gain was noted in this series, patients felt that the addition of stereoscopic visualization improved their overall understanding. It therefore potentially increases patient confidence in treatment decisions.

**Supplementary Information:**

The online version contains supplementary material available at 10.1007/s00701-023-05512-x.

## Introduction

### The role of the surgical informed consent

The informed consent conversation is obligatory prior to a surgical intervention in the clinical routine and an irreplaceable component of patient-centered care. Every physician is morally and legally obligated to inform the patient before a medical intervention about the patient-specific anatomy, symptoms and prognosis of the disease, and the steps and risks of the planned surgery. Informed consent is also of major importance when it comes to respecting the patient’s autonomy [15]. In this context, Park et al. outlined that a better understanding of the patient’s individual illness and the upcoming surgery can lead to a greater sense of control and autonomy [25]. What is everyday routine for physicians can be a life-changing event for patients. Proper informed consent can alleviate patient anxiety and lead to more realistic expectations, as misperceptions can be identified and corrected [24]. Another aim of the discussion with the patient about the operation and the possible complications is to prevent a possible lawsuit [19]. It has been found that miscommunication between the patient and the physician is more likely to result in litigation than technical failure [19]. In addition, miscommunication can have a negative impact on the patient’s trust in the treating physician. Trust is important for the patient-doctor relationship and can positively influence the treatment results [30]. As laypersons, it is often difficult for patients to fully understand the content of medical discussions with their doctors; thus, implementation of newer technologies is welcome to improve the informed consent.

### New paths of surgical informed consent

Innovations in medicine and technology enable increasingly complex diagnostics and interventions [36]. This enhances the quality of patient care but simultaneously raises the difficulty of explaining the complex surgery in connection with the anatomic conditions to the patients. Therefore, it is of great importance to improve the quality of surgical informed consent in general, for which more personalized approaches are warranted. This is particularly true when it comes to neurosurgical interventions. Neurosurgical operations are intricate, and the complexity of brain anatomy is difficult to understand even for specialists, hence demanding the most realistic imaging available [9, 34]. A systematic review by Shlobin et al. highlighted the great need for improvement in patient education in neurosurgery, where low patient comprehension and poor quality of the present information sources were demonstrated [31]. A recent review has analyzed the baseline patient recall after conversations with neurosurgeons in particular and the effects of interventions to improve patients’ comprehension [33]. In this study, various intervention modalities such as illustrations and interactive checklists were identified that led to an improvement in patients’ knowledge [33]. Here, it has been shown that modern technologies can improve patient education. Leclercq et al. already noted back in 2010 that interactive computer programs increased the quality of the surgical informed consent [19]. For instance, a systematic review by Schenker et al. in 2010 displayed an improvement in patients’ understanding using multimedia interventions [28]. However, most of the studies in this review examined only the use of non-personalized videos by physicians who talked about the potential risks of the various interventions. The use of other modern technologies that allow a more personalized visualization of the patient’s pathology, such as three-dimensional (3D) printing and virtual reality (VR), has increased in the context of informed consent [13, 17, 20, 26, 38]. VR is a convenient way to visualize the patient’s anatomy and the doctors’ actions during an intervention [3, 6, 26]. In this regard, Perin et al. showed that the patient-doctor relationship can be improved through the use of VR with the visualization of the patient’s own images. Here, the patients’ comprehension capability could be improved without increasing their anxiety at the same time [26]. On the other hand, the 3D prints proved to be anatomically accurate and helpful in patient consultation [17]. A positive effect on the patients’ knowledge by 3D printing was reported by Yoon et al. in patients with lung cancer prior to surgical resection [38]. However, 3D prints are expensive and time consuming [35]. Stereoscopic visualization could be a possible alternative as it is cheaper and easier to implement into clinical routine. This might be an advantage over the so far better-analyzed 3D printing [14]. A stereoscopic visualization creates a perception of depth by projecting a slightly offset image onto each eye and could also be used to demonstrate patient images [23]. Stereoscopic imaging in the form of three-dimensional presentation of computed tomography (CT) and magnetic resonance imaging (MRI) has already found its way into modern medicine in teaching and preoperative planning [1, 9, 11, 16, 29]. However, an evaluation of the stereoscopic visualization of patients’ own images within the surgical informed consent has not yet been performed. One can assume that this simple but personalized add-on during the patient consent conversation may be sufficient to improve patients’ understanding. Thus, our goal in this study was to investigate the potential benefits of additional stereoscopic visualization of patient-specific imaging during the surgical informed consent conversation prior to a brain tumor resection at our neurosurgical department.

## Methods

### Study protocol

The study was performed in accordance with the ethical standards of the Declaration of Helsinki 1964. The ethics committee of Charité – Universitätsmedizin Berlin approved the present randomized prospective study (EA2/019/18). The study is registered in the German Clinical Trials Register (DRKS00014930). Informed consent was obtained from all individual participants included in the study.

**Fig. 1:**
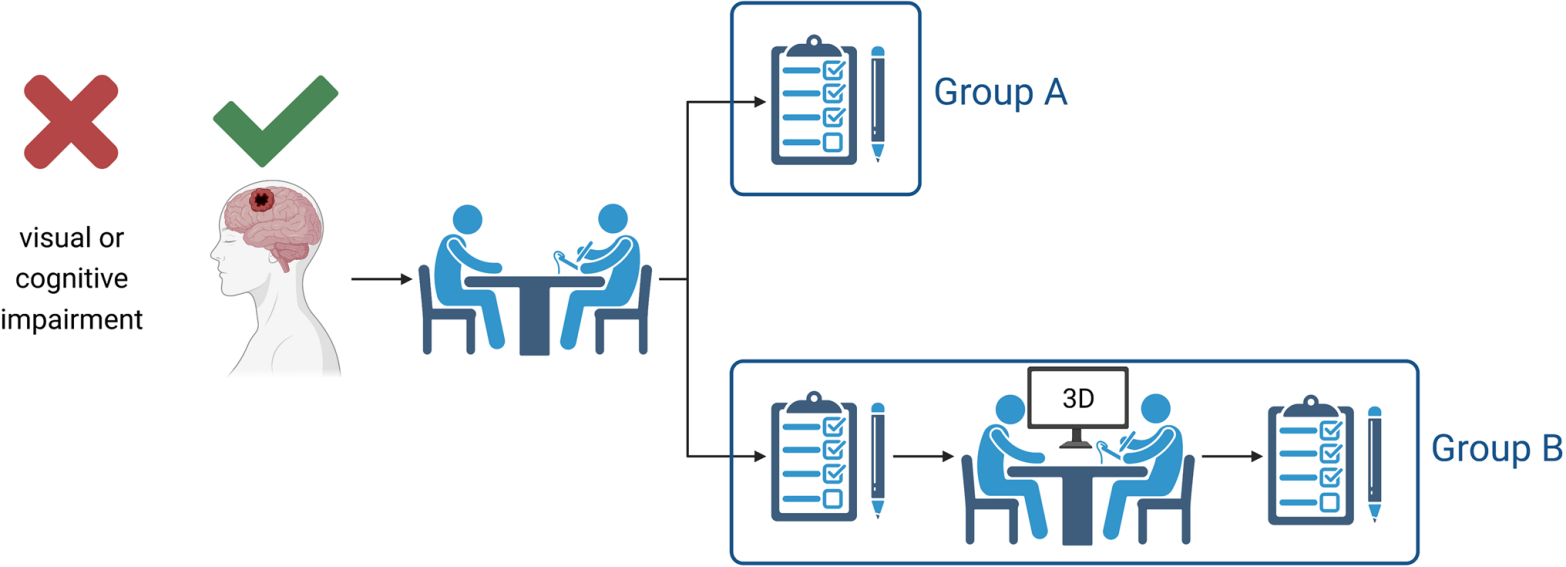
Flowchart of the study design. Group A received the surgical informed consent in a conventional manner, while group B received an additional stereoscopic visualization with a second consent conversation. Created with BioRender.com

Patients who were scheduled for a brain tumor surgery at our department between June 2018 and January 2019 were screened for this study. Patients with impending brain tumor surgery were included regardless of tumor type, location, or prior treatments. The primary exclusion criteria were cognitive or visual impairments. Patients’ capacity to give consent was critically assessed by the physician who conducted the routine consent interview, independent of the study. Only patients capable of signing their own consent in routine clinical practice were considered for this study. All eligible participants were first tested for stereopsis with the Frisby Pocket Stereotest™. All patients gave their surgical informed consent to a medical doctor from our department after being informed about the procedure in a conventional way prior to the randomization. Then, the participants were randomized into group A without any further interventions or group B with the additional stereoscopic MRI visualization of their own pathology including a second explanation of the planned surgical intervention by the same physician who had performed the routine consent (Fig. 1). The stereoscopic imaging 3D spatial mouse was technically controlled by a consistently researching medical student who was guided by the physician during the interview to present the required image in the correct format. The 3D images were explained by the physician in accordance with the routine informed consent interview. The visualization with the explanation took about 20 min. After each informed consent, the participants filled in a questionnaire, which resulted in group B completing a total of two questionnaires (Fig. 1).

### Questionnaires

The patients were asked to give feedback about the process of the informed consent with their subjective opinion using a 5-point Likert scale in the questionnaires. The other questions were focused on the anatomical location of the tumor, the surgical procedure, and the possible complications (Supplemental Questionnaire). The objective understanding was measured as the number of correct answers per case for the anatomical location of the patient’s tumor, the complications mentioned, and the surgical steps. A maximum of 4 points could be obtained for the location of the tumor and a maximum of 5 points for the surgical steps. For the nearby anatomical structures and complications, one point was awarded for each designation. For the complications, the highlighted most relevant risks per surgery were around 20 items focusing on the anatomical region of the tumors. In the results, the location and structures are summarized under the term “Anatomy,” while the surgical steps and complications are grouped under the term “Operation.”

In the stereoscopic group, we additionally conducted two semi-structured qualitative interviews after the completion of both informed consent talks to identify general topics in patient perception of diagnostic medical imaging. Participation in the interview was offered to the patients on a voluntary basis. The main survey topics were surgical risk, tumor size and location, and surgical approach. Interview transcripts were pseudonymized before analysis.

### Image processing and visualization

**Fig. 2:**
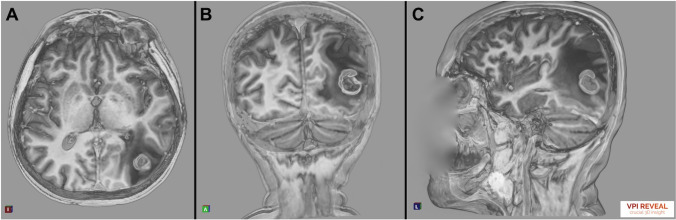
Representative screenshots from the demonstration of metastasis from bronchial carcinoma using the VPI Reveal system. The location is on the left parietal lobe measuring 19 × 18 mm. The screenshots (**a** axial plane, **b** coronal plane, **c** sagittal plane (the face is covered for privacy protection) are from a demonstration video (Supplemental Video 1)

Using the program VPI Reveal, version 1.5 (Vesalius Perfectus International BV, Eindhoven, the Netherlands), three-dimensional (3D) volume rendering as a display of the MRI was performed (Fig. 2). VPI Reveal is a proprietary software based on an in-house–developed 3D volume rendering technology. It is using raytracing technology and dedicated image post-processing to facilitate optimal stereoscopic 3D display (holographic 3D). The DICOM images were imported from the hospital’s picture archiving and communication system (PACS) into the VPI Reveal software. A three-dimensional model was generated from the software. Subsequently, we could modify the visualization settings to improve the quality. For this purpose, each Hounsfield unit could be assigned with a specific gray value or a color. An individual grayscale was chosen. The contrast and transparency of the images were adjusted, so that an individual 3D model could be generated. This process required approximately 3 min per case. The study was conducted at two different hospital sites. The research team used either a high-resolution liquid crystal display (LCD) monitor (Hyundai S465D; 3D LCD monitor, 46 in.; resolution: 1920 × 1080; 60 Hz) or a high-resolution light-emitting diode (LED) monitor (LG 55LA6208; 3D LED monitor, 55 in.; resolution: 1920 × 1080; 200 Hz MCI) for 3D display. For image navigation, we used a 3D mouse (3Dconnexion SpaceMouse Compact). Commercially available polarized 3D glasses by Hyundai and LG were used. The VP Reveal was running on a Dell Inc. Precision 7720 computer with an Intel Core™ i7-7920HQ (3.10 GHz) central processing unit and an NVIDIA Quadro P4000 graphics card [29].

### Statistical analyses

The statistical analyses were performed by using SPSS version 25 (International Business Machines Corporation (IBM), Armonk, NY). The graphs were visualized with GraphPad Prism version 9 (GraphPad Software, San Diego, CA) and edited with PowerPoint version 16 (Microsoft, Redmond, WA). Data and tables were managed with Excel version 16 (Microsoft, Redmond, WA).

Descriptive statistics were given as median with limits of the interquartile range (IQR) [25th–75th percentile]. For the statistical evaluation, the results of group A and the second questionnaire (after stereoscopic visualization) of group B were compared by using the Mann–Whitney *U* test. To study the two groups in terms of the distribution of age, gender, and education level, the chi-square test was applied (Table 1). For group B, Wilcoxon’s signed-rank test was used to compare the results before and after stereoscopic visualization as intra-rater analysis. All tests were two-sided, and *p* < 0.05 was considered to indicate statistical significance. Since this is an exploratory analysis, no adjustments were made for multiple testing.

**Table 1:** Comparison of group A and group B on mean age, percentage of patients under and over 65 years, gender distribution, level of education, and type and location of tumor (age: Mann–Whitney *U* test; others: chi-squared test)

	Group A (*n* = 15)	Group B (*n* = 12)	*p*
Age (median [IQR])	61 [51–69]	48 [39.5–63.75]	0.126
Age (< 65/ ≥ 65)	60%/40%	83.3%/16.7%	0.187
Gender (female/male)	46.7%/53.3%	75%/25%	0.137
Education level (college/high school/no information)	13.3%/73.4%/13.3%	41.7%/50%/8.3%	0.248
Tumor type			0.879
Meningioma WHO grade I	6 (40%)	3 (25%)	
Brain metastasis	4 (26.7%)	4 (33.3%)	
Malignant glioma WHO grade III/IV	3 (20%)	3 (25%)	
Pituitary adenoma	2 (13.3%)	2 (16.7%)	
Tumor location			0.399
Frontal lobe (left/right)	4 (26.7%)/2 (13.3%)	2 (16.7%)/2 (16.7%)	
Parietal lobe (left/right)	3 (20%)/1 (6.7%)	1 (8.3%)/0	
Occipital lobe (left/right)	0/2 (13.3%)	0/1 (8.3%)	
Temporal lobe (left/right)	1 (6.7%)/0	3 (25%)/1 (8.3%)	
Pituitary gland	2 (13.3%)	2 (16.7%)	

## Results

### Patient cohort

**Fig. 3:**
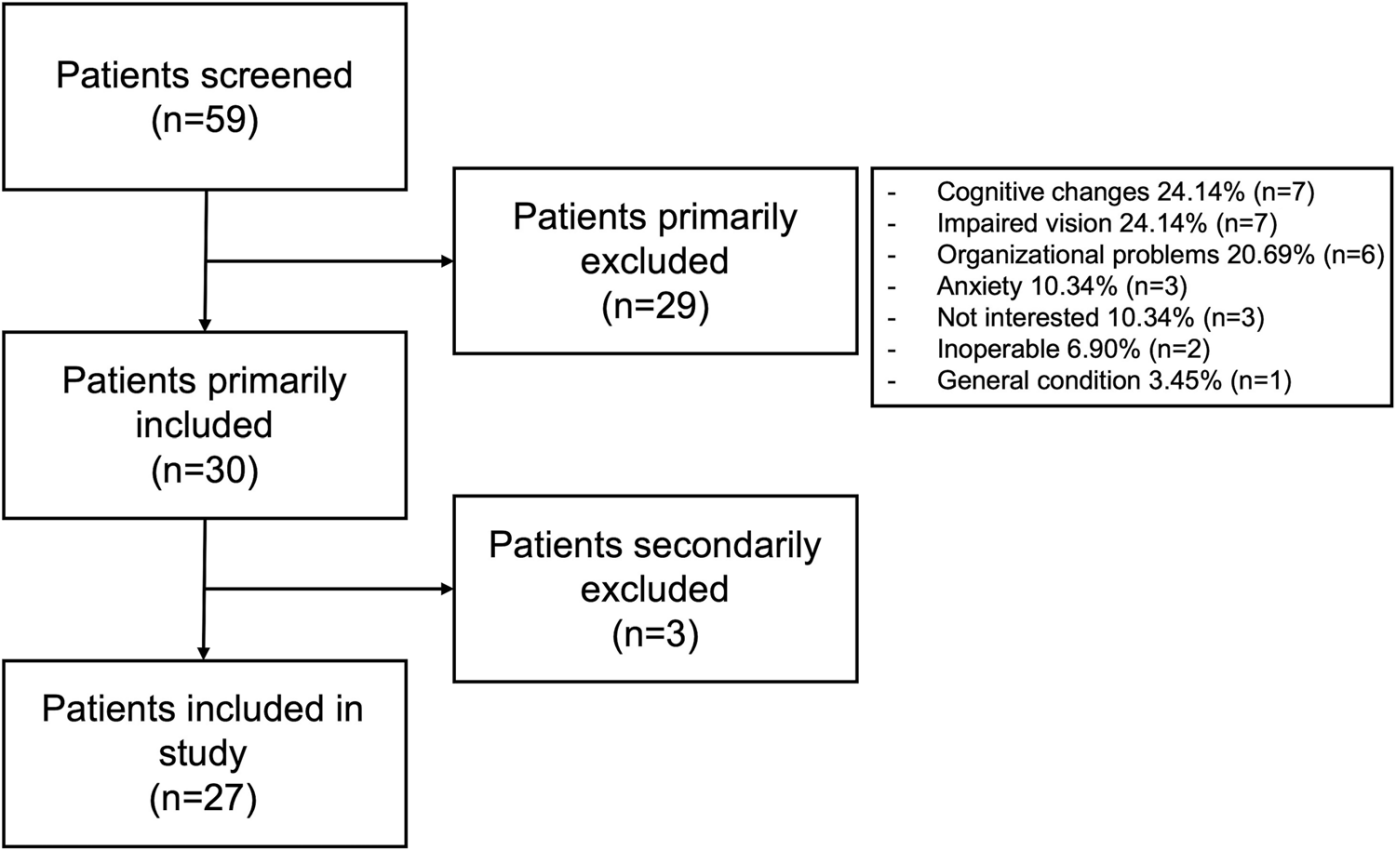
Flowchart outlining the inclusion process with a list of reasons for exclusion. The secondary exclusion was due to organizational issues

A total of 59 patients were screened for this study. After the initial screening, 29 patients were excluded, mostly due to dementia and visual impairment. Three additional patients were excluded from the study by secondary exclusion due to organizational issues (Fig. 3).

The final number of patients enrolled in the study was 27, of whom 16 (59.3%) were female and 11 (40.7%) were male. The median age was 61 [interquartile range (IQR) 43–68]. Among the participants, 7 (25.9%) were college graduates and 17 (63%) were high school graduates; no information was provided by 3 (11.1%) individuals. Of all included participants, 15 were randomized to group A and 12 to group B without any statistically significant differences among compared patient characteristics (Table 1). The relative frequencies of tumor pathology of the two groups were similar, with nearly half of the patients in group A having benign tumors (including meningiomas and pituitary adenomas) (53.3%), compared with 41.7% in group B. Accordingly, malignant tumors (including brain metastases or gliomas) were slightly more common in group B (58.3%) than in group A (46.7%). There was also no statistically significant difference between the tumor locations of the two groups. On closer inspection, more tumors were found in the parietal lobe in group A (26.7%) than in group B (8.3%) and in the temporal lobe in group B (33.3%) than in group A (6.7%).

### Subjective evaluation

**Fig. 4:**
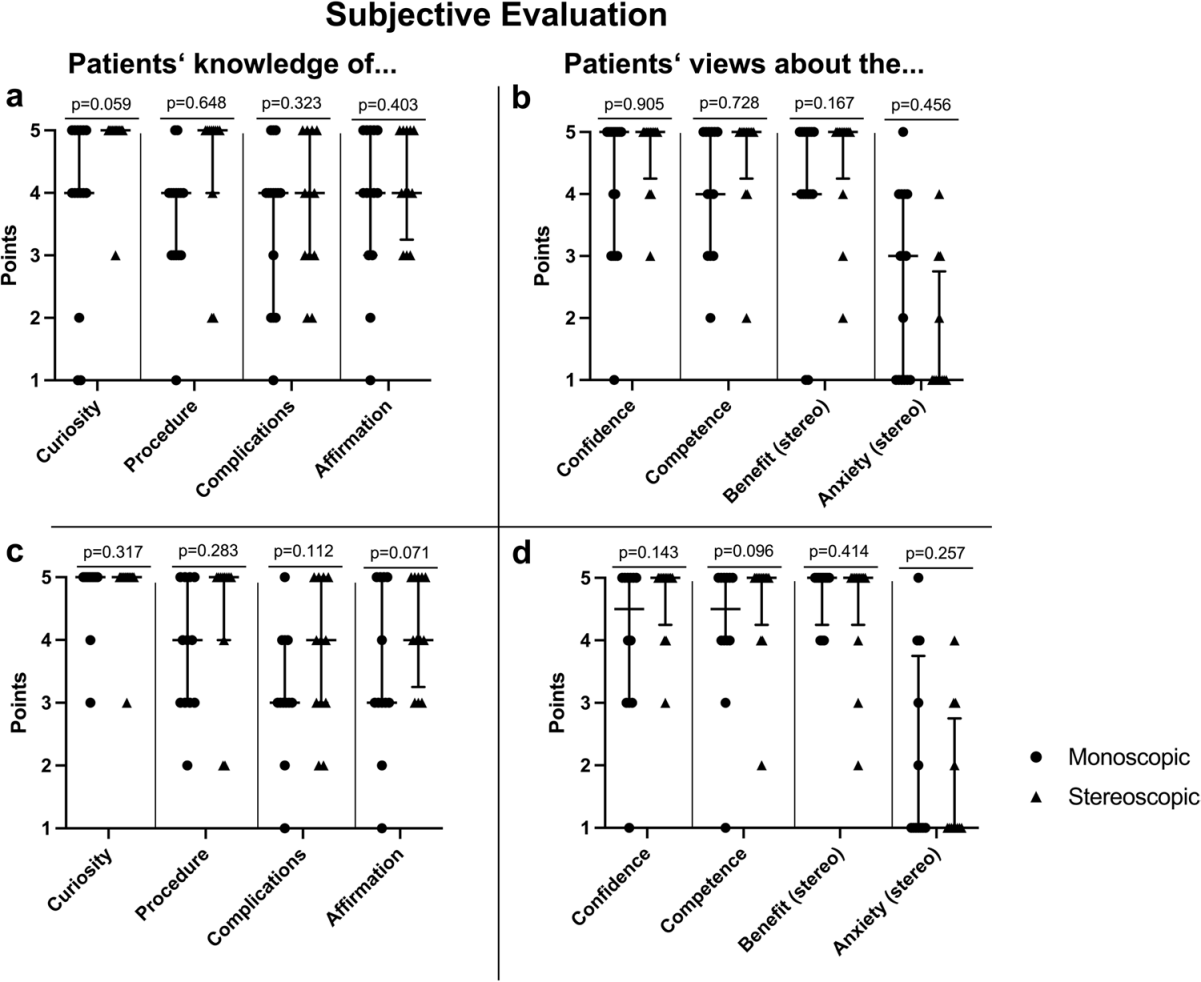
**a** Comparison of group A (*n* = 15; dots left in each column; monoscopic) with group B (*n* = 12; triangles right in each column; stereoscopic) with a focus on the surgery- and patient-doctor relation and stereoscopy-related subjective categories. (**b**) Mann–Whitney *U* test. **c**, **d** In-group comparison of the 12 participants of group B for the same 12 categories separately after monoscopic (dots left in each column; monoscopic) and after stereoscopic MRI visualization (triangles right in each column; stereoscopic) as intra-rater analysis (Wilcoxon signed-rank test). The dots and triangles represent the points awarded by each participant for the various categories. Additionally, the lines represent the median and the interquartile range [25th–75th percentile]. Curiosity was defined as the patient’s general interest in information about the upcoming surgery. The procedure represents the subjective knowledge about the upcoming surgery, and complication constitutes the subjective knowledge about the possible complications. Affirmation refers to the patient’s encouragement through this type of informed consent to have the procedure performed. Confidence represents the trust in the department engendered by this type of informed consent. Competence portrays the competence of the department conveyed by this type of informed consent. Benefit was defined as the (possible) advantage of stereoscopic visualization. Anxiety constitutes the (possible) anxiety caused by the stereoscopic visualization

The analysis of the subjective evaluation was performed in order to evaluate patients’ personal perceptions of the informed consent process. In the first questionnaire, participants from group A appeared to have a strong interest in information about their upcoming surgery. The manner in which the consent process was carried out was assessed as very confidence inspiring. Patients also indicated that they were well informed about the upcoming surgery and the possible complications. The participants from group A rated the increase in potential anxiety as medium and the potential benefit as high prior to a stereoscopic visualization. The results for group B showed that after the stereoscopic visualization of the tumor; the patients showed no anxiety at all. The patients rated the new imaging method as helpful. Like in group A, participants felt well informed about the possible complications and confident about the procedure. In the comparative analysis of group A vs. group B, there was no statistically significant difference in the understanding of the procedure. Overall, there were no statistically significant differences between the conventional and stereoscopic groups in terms of subjective evaluation (Fig. 4a, b).

Further analysis within group B to compare status before and after stereoscopic visualization showed no statistically significant increase in subjective knowledge of the upcoming surgery or in its possible complications. Subjective assessment of benefit after 3D presentation corresponded with expected benefit before stereoscopic visualization. This also applies to the possible fear, which was estimated low prior to the presentation and was confirmed as low after the 3D presentation. Overall, the intra-rater analysis prior to and after the stereoscopic visualization provided comparable results (Fig. 4c, d).

### Objective analysis

The questions about exact anatomical conditions and the course of the surgical procedure with possible complications were evaluated to determine the objective benefit of the stereoscopic visualization during informed consent. Patients from group A and group B could both not well identify the location of their own tumor and related structures, while all patients were able to name the steps of the surgery and possible complications equally well. When patients were asked to recall the complications, group A and group B listed both a median of 2 items. Overall, the participants did not objectively possess statistically significantly more knowledge about their own tumor anatomy or the surgical procedure after the stereoscopic add-on, although a positive trend could be observed.

**Fig. 5:**
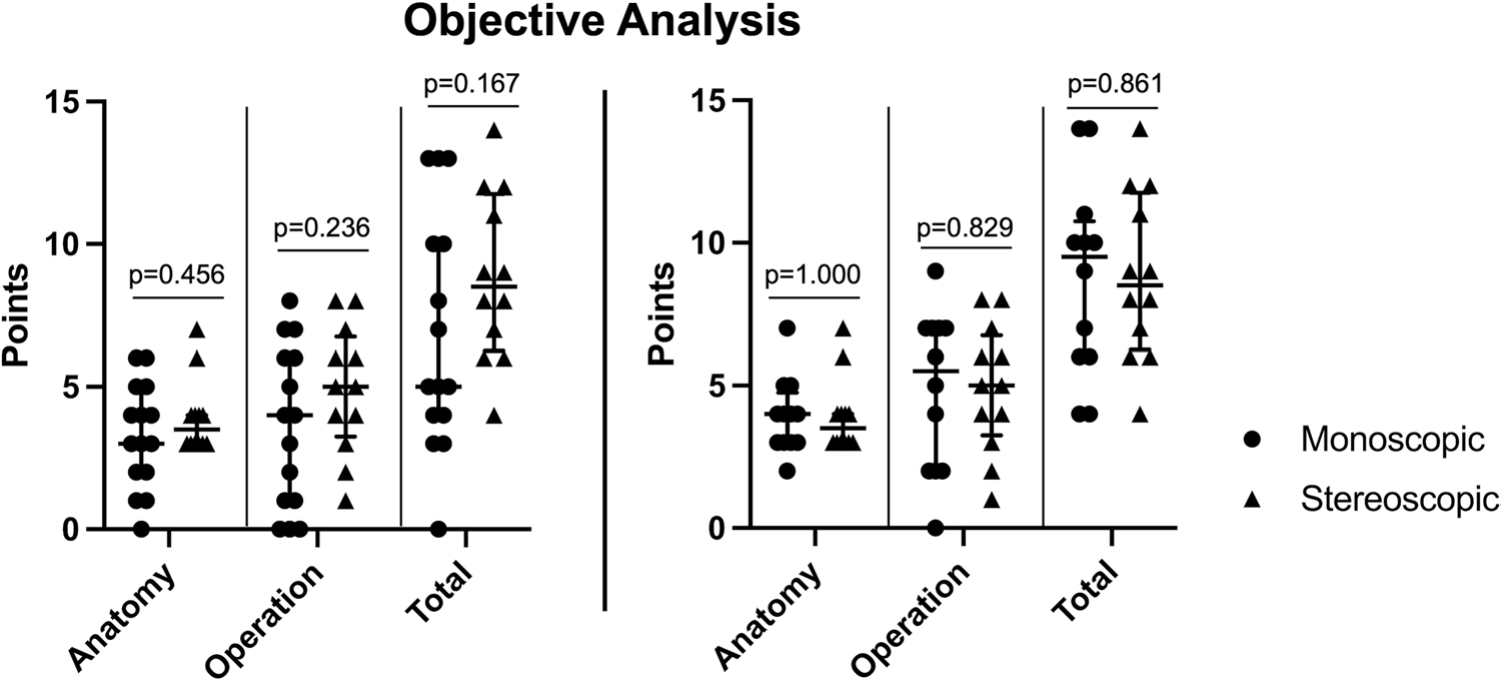
Left: Comparison of group A (*n* = 15; dots left in each column; monoscopic) and group B after the second consent conversation with stereoscopic imaging (*n* = 12; triangles right in each column; stereoscopic) regarding the points achieved in terms of anatomy, operation, and as a total (Mann–Whitney *U* test). Right: In-group comparison of the 12 participants of group B after monoscopic (dots left in each column; monoscopic) and after stereoscopic MRI visualization (triangles right in each column; stereoscopic) regarding the points achieved in terms of anatomy, operation as intra-rater analysis (Wilcoxon signed-rank test). The dots and triangles represent the achieved points by each participant for the various categories. Additionally, the lines represent the median and the interquartile range (25th–75th percentile)

The intra-rater analysis within group B (before and after the stereoscopic visualization) demonstrated that comprehension was not improved by the addition of stereoscopic visualization significantly (Fig. 5). These patients were able to recall a comparable number of items before and after the additional consent conversation.

### Subgroup analyses

We performed further subgroup analyses combined for both monoscopic groups (group A + group B before stereoscopic visualization) with a focus on education status and age. The comparison between college graduates and high school graduates did not reveal any statistically significant difference in the objective evaluation (total points achieved: high school graduated (*n* = 17): median 6 [IQR 4–10]; college graduated (*n* = 7): median 10 [IQR 8–12.5]; *p* = 0.087). A statistically significant difference was observed between patients < 65 years of age (*n* = 19) and those ≥ 65 years of age (*n* = 8) in terms of total points achieved in the objective evaluation (total points achieved: < 65 years: median 10 [IQR 7–13]; ≥ 65 years: median 4 [IQR 3.25–5]; *p* < 0.001).

### Qualitative interviews

Two of the 12 patients in the stereoscopic group consented to a qualitative post hoc interview after completing both informed consent talks and questionnaires: one diagnosed with suspected pituitary adenoma and one with suspected sphenoid wing meningioma. In both cases, prior exposure to the imaging modality used in the study affected comprehension during informed consent; one patient had MRI scans before, and the other was familiar with it through popular culture. Compared to conventional informed consent based on selected CT or MRI images, the stereoscopic visualization was perceived to support a better understanding of anatomical relationships because it provided relevant context information. The sagittal view in particular was highlighted as the most intelligible depiction of the neurocranium. Both interviewees used it for relating to external landmarks of the head, such as the nose and ears, in order to better situate intracranial processes. The question of visual likeness between patient and image also became more relevant in a direct comparison of 3D reconstruction and 2D sectional imaging. One interviewee stated that the unfamiliar esthetics of sectional imaging were easier to accept as diagnostic truth than the immersive close-up of the individual neuroanatomy in 3D. They perceived the volumes of radiological thin layer imaging as rather “unprocessed” raw data, while the more accessible 3D reconstruction already served as an “interpretation.” However, both patients recommended the stereoscopic visualization for routine clinical application and asked for more and earlier technology transfer in the realms of doctor-patient communication.

## Discussion

In this study, we analyzed the potential benefits of an additional personalized 3D stereoscopic visualization of patients’ individual imaging during the informed consent procedure in 27 patients with brain tumors prior to a neurosurgical resection. Although we did not observe any statistically significant subjective or objective benefits of this additional stereoscopic visualization, the patients expressed a high level of interest in the new imaging modality reflecting the potential benefit in the future. It is important to note that patients’ anxiety was not increased by the more realistic presentation of their own imaging.

### Surgical informed consent in neurosurgery

The field of neurosurgery and the specific anatomy of the brain can present a great challenge even for experts. The surgical procedures in the brain are particularly complex, and it is very difficult for patients to understand them even in lay terms [34]. Therefore, accurate imaging techniques and their presentation during informed consent are of major importance to support patient understanding. A systematic review by Dicpinigaitis et al. analyzed preoperative information sessions for patients undergoing neurosurgical procedures. They concluded that educational interventions, such as VR and 3D printing, but also text-based or video-based, lead to improved patient knowledge and satisfaction [5]. As an example, the use of VR during informed consent conversation improved patients’ understanding of their condition without increasing anxiety in patients with intracranial tumors in a previous study [26]. Kim et al. analyzed the role of patient-specific 3D printing in neurosurgical patients with an unruptured cerebral aneurysm [17]. Their findings demonstrated enhanced understanding in the 3D-printing group, which highlights the potential benefit of such technical improvements in neurosurgical practice [17]. However, as previously mentioned, 3D printing is expensive and time-consuming [35]. In this regard, a study by Lee et al. showed that neurosurgical patients showed improved understanding after receiving two informational booklets, three videos, and active comprehension support [20]. A week later, after a new 15-min interview, the patients from the intervention group achieved greater comprehension than the ones in the comparison group [20]. However, implementing additional methods of patient education in the busy clinical routine can be difficult as time is quite limited. Also, that information lacks personalization. We were striving to investigate a realistically implementable intervention, as stereoscopic images of already performed MRI scans are easy to prepare. Thus, we analyzed the potential benefit of stereoscopic visualization of patients’ individual MRI images to explain the disease and surgical procedure in a personalized manner.

The subjective analysis showed that the patients already indicated a high level of understanding for the upcoming procedure and possible complications after the conventional patient consent conversation. However, these subjective statements could not be confirmed objectively since the patients did not perform well on the anatomical and procedural questions. In our study, adding stereoscopic display of imaging data did not improve patient competency. Likewise, Agarwal et al. did not observe any improvement in patient knowledge in another neurosurgical study, where patients with cerebral aneurysm in the intervention group received a presentation with text and visual graphics [2]. In addition, a systematic review by Glaser et al. in 2020 showed that audiovisual intervention in the form of visual or audiological recordings improved patients’ comprehension in only about half of the studies examined (56% of 27 trials) [7]. Surprisingly, in our study, even an additional stereoscopic visualization and a longer interaction with the treating physician did not lead to a significant improvement in naming anatomical relations or surgical procedure steps. However, this could be also due to the low number of participants, as we observed a trend as seen in the confidence intervals. To account for the heterogeneity of patients in terms of tumor type and location, among other factors, before-and-after analysis in the same patient was very essential. Importantly, we also found no significant differences between before and after the additional stereoscopic visualization in the intra-rater analysis of these patients in group B, but here, the number of participants was even smaller, which lowers the power of the statistical analysis. On the other hand, one can also assume that the patient is quickly overwhelmed with information and a further increase in understanding might not be achievable. This could be due to the many technical terms that are inevitably used and the amount of new information. In this context, Saigal et al. conducted a study where patients watched a video about incidence rates of 11 possible complications as an addition to the informed consent for spinal deformity surgery [27]. The patients could recall only 45% of the video directly after it. The value even decreased to 18% after a time period of 6 to 8 weeks. Similar to our study, Huber et al. also failed to demonstrate an increase in knowledge with the use of multimedia support in patients undergoing radical prostatectomy [10]. Explicitly related to the listed risks, there was no difference between the group that received multimedia support and the group that received conventional education in the study [10]. Unfortunately, the number of mentioned risks was not mentioned in the study. In a study by Krupp et al., neurosurgical patients undergoing brain surgery and spine surgery were able to recall in median five and four risks out of 32 and 25 assumed risks, respectively [18]. However, the patients in our study could name only two risks in median. In our interviews, the most relevant risks were explained to the patients using around 20 items based on the anatomical region. A comparison between the complexity of the interventions in these two studies was not possible. However, while the proportion of college graduates was similar (24% vs. 26%), the patients in the study by Krupp et al. were younger (52 years vs. 61 years in median in our study). This could be a possible explanation for the better performance of the patients in Krupp et al., since we also observed a significantly better performance on the questionnaire by the younger participants in our study. Our findings were consistent with the study of Lo et al., who also demonstrated significantly better recall in patients under 65 years of age in comparison with those at or over 65 years of age [21]. Interestingly, we did not find statistically significant differences according to the different educational levels of the participants in our cohort. When looking at the medians, however, a trend could be seen. This would be consistent with the findings of Crepeau et al., that demonstrated how orthopedic patients with at least a college degree achieved a significantly higher score on a test prior to surgery than patients without higher education [4]. It is important to note that we did not perform neurocognitive testing to assess patients’ IQ or higher cognitive functions in this regard.

With regard to patient and tumor characteristics, we did not find significant differences between the two groups. Tumor locations differed slightly but did not reach significance. Strikingly, meningiomas as extra-axial tumors were slightly more frequent in group A (40%), but since the overall proportion of benign and malignant entities was similar in both groups, we consider that the two groups were well comparable. Importantly, in our study, the only difference between the two consents was the visualization technique of the personalized MRI, while the content and the doctor in charge remained the same for each patient. As non-medical laypersons have difficulty understanding two-dimensional (2D) cross-sectional images of intracranial topography, patients seem to connect more easily to the somewhat more lifelike stereoscopic visualization of their own neuroimaging data. One could argue that surgeons may use stereoscopic 3D for better anatomical preparation while patients have other, more general primary concerns such as the confirmed existence, location, and size of a brain tumor as the individual interviews have shown. Interestingly, the patients preferred the sagittal view while physicians tended to prefer the axial view. Perhaps the participants were able to better assess the position and size relationships in the profile of the face presented in the sagittal view. This should be considered for future investigations.

New possibilities of video recording techniques also enable other innovative ways of informing patients. For instance, it is now possible to generate 360-degree surgical videos that could be integrated in the surgical informed consent process. These videos could also be annotated if needed as Johnson et al. recently did to educate patients for external beam radiation therapy [13]. Patients in this study noted a possible improvement in knowledge about their upcoming intervention. In order to invent new digital information tools, it could be also helpful to resort to the concept of participatory design where patients are integrated in the development of newer approaches [8]. Taking the individual needs of the patients into account may improve the success rate of new media.

### The patient-doctor relationship

As mentioned above, the patient consent conversation influences patients’ autonomy and confidence [15]. Therefore, one of the most important aspects of assessing patient consent is its impact on the patient-doctor relationship. However, this poses a problem as it is difficult to quantify. We used a 5-point Likert scale, as this self-report scale is an essential component in subjective assessment [12]. Patients were asked to rate, among other things, their confidence in the neurosurgeons and evaluation of the department’s competence. Here, our study indicated a good patient-doctor relationship, which was reflected by the high values given by the patients. Marone et al. concluded that the use of 3D printing in patient education has shown to improve the patient-doctor relationship [22]. An objective method of measurement was not provided by the authors, but they describe 3D printing as a communicative tool for obtaining informed consent. The second part of the review mentioned before by Shlobin et al. revealed that patients prefer a digital form of education, for example, VR over conventional informed consent [32]. However, this and similar types of three-dimensional presentations can also be considered emotionally confronting in the early stage of treatment [32]. In this one study, the more realistic representation provided by a personalized 3D print was described as frightening [37]. Fortunately, this finding could not be reproduced in our study. It should be noted that only 3 of 59 (5.1%) screened patients reported anxiety as a reason for non-participation and none of the patients interrupted the study. Importantly, participants in our study did not report increased anxiety after stereoscopic visualization.

### Limitations

The major limitation of our study is the low number of participants. A further drawback of the study design is that we did not perform a 1:1 randomization to either conventional or stereoscopic visualization, as we wanted to avoid any legal issues to due lack of conventional patient consent. In this context, we also observed an imbalance in age and education between both groups; however, this was not statistically significant. One minor pitfall was that the imaging navigation had to be controlled by a study staff member, as the 3D mouse is difficult to use. However, during the implementation of the study, patients were impressed by and appreciated the modern form of individual visualization and the reported evaluation provides the first insights into using stereoscopic visualization in neurosurgical consent conversations for brain tumor patients.

## Conclusion

In comparison with conventional preoperative patient information based on 2D sectional imaging, stereoscopic visualization of individual MRI during informed consent conversation did not improve the knowledge of the patients in our study. However, stereoscopic visualization seemed to positively impact the patient-doctor communication by supporting the patients’ visual imagination with a better description of the procedural steps. We observed a high demand and acceptance of stereoscopic imaging among patients. Therefore, our findings support the further implementation of visual media in the informed consent procedure, such as 360-degree videos, interactive apps, or other mixed reality applications, in the future.

## Supplementary Information

Below is the link to the electronic supplementary material.

Supplementary file1 (MOV 47162 KB)

Supplementary file2 (PDF 46 KB)

## Data Availability

The original material can be requested from the corresponding author and may be supplied anomously.

## Abbreviations

CT: Computed tomography
DICOM: Digital imaging and communications in medicine
IQR: Interquartile range
LCD: Liquid crystal display
LED: Light-emitting diode
MRI: Magnetic resonance imaging
PACS: Picture archiving and communication system
VR: Virtual reality
2D: Two-dimensional
3D: Three-dimensional
