# Supplementary material for: Personalized surgical informed consent with stereoscopic visualization in neurosurgery—real benefit for the patient or unnecessary gimmick?
Source: Acta Neurochir (Wien). 2023 Feb 28;165(4):1087–98. doi: 10.1007/s00701-023-05512-x (PMC10068664; doi:10.1007/s00701-023-05512-x)
Supplement: Supplementary file 2 — Supplementary file2 (PDF 46 KB) [file 701_2023_5512_MOESM2_ESM.pdf]

ChariteCentrum für Neurologie, Neurochirurgie und Psychiatrie

Charité | Campus Mitte | 10117 Berlin

Neurochirurgische Klinik und Hochschulambulanz
Direktor: Prof. Dr. med. Peter Vajkoczy
Tel. (030) 450 560002
Fax (030) 450-560900
http://neurochirurgie.charite.de

Datum: 01.06.2018

Surgical Informed Consent Study

Questionnaire: mono / *stereo*

Name: Date:

Previous Surgeries: Occupation:

1. Please indicate the location of your tumor (most applicable please mark with a cross, 3 crosses!):

   - ☐ right
   - ☐ left
   - ☐ front
   - ☐ central
   - ☐ back
   - ☐ superficial
   - ☐ deep

2. Please indicate the height of your tumor in relation to the nose-ear line (most likely to apply please tick):

   - ☐ above
   - ☐ at the level of the nose-ear line
   - ☐ below

3. Please name one or more important brain structures that are located near the tumor:

   ____

   ____

4. Please describe the necessary steps of the intervention in your own words:

   ____

   ____

   ____

5. Please name the risks involved in your procedure:

   ____

   ____

   ____

6. Please answer the following questions on a scale from 1 (strongly disagree) to 5 (strongly agree):
   a. Would you like to know the basics of what exactly happens during your procedure?

      1 2 3 4 5

   b. After being informed, can you clearly imagine what will be done during your procedure?

      1 2 3 4 5

   c. Can you assess the possible complications of your procedure in terms of their importance and likelihood?

      1 2 3 4 5

d. Do you find this type of information confidence-building?

1 2 3 4 5

e. Does this type of information convey the department's expertise?

1 2 3 4 5

f. Did this type of information strengthen your consent to the procedure?

1 2 3 4 5

g. Do you think a 3D representation would have been helpful in your understanding of the procedure? / *Did you find the 3D representation helpful in your understanding of the process?*

1 2 3 4 5

h. Do you think a 3D representation would have increased your anxiety about the procedure? / *Did you find the 3D representation increased your anxiety about the procedure?*

1 2 3 4 5
